# Production and partial purification of membrane proteins using a liposome-supplemented wheat cell-free translation system

**DOI:** 10.1186/1472-6750-11-35

**Published:** 2011-04-11

**Authors:** Akira Nozawa, Tomio Ogasawara, Satoko Matsunaga, Takahiro Iwasaki, Tatsuya Sawasaki, Yaeta Endo

**Affiliations:** 1 Cell-Free Science and Technology Research Center and the Venture Business, Laboratory, Ehime University, 3 Bunkyo-Cho, Matsuyama, Ehime 790-8577, Japan; 2 RIKEN Systems and Structural Biology Center, 1-7-22 Suehiro-cho, Tsurumi-ku, Yokohama, Kanagawa 230-0045, Japan; 3 Proteo-Medicine Research Center, Ehime University, Toon, Ehime 791-0295, Japan

## Abstract

**Background:**

Recently, some groups have reported on cell-free synthesis of functional membrane proteins (MPs) in the presence of exogenous liposomes (liposomes). Previously, we reported synthesis of a functional AtPPT1 plant phosphate transporter that was associated with liposomes during translation. However, it is unclear whether or not lipid/MP complex formation is common to all types of MPs in the wheat cell-free system.

**Results:**

AtPPT1 was synthesized using a wheat cell-free system with or without liposomes. AtPPT1 synthesized with liposomes showed high transport activity, but the activity of AtPPT1 synthesized without liposomes was less than 10% activity of that with liposomes. To test whether co-translational association with liposomes is observed in the synthesis of other MPs, we used 40 mammalian MPs having one to 14 transmembrane domains (TMDs) and five soluble proteins as a control. The association rate of all 40 MPs into liposomes was more than 40% (mean value: 59%), while that of the five soluble proteins was less than 20% (mean value: 12%). There were no significant differences in association rate among MPs regardless of the number of TMDs and synthesis yield. These results indicate that the wheat cell-free system is a highly productive method for lipid/MP complex formation and is suitable for large-scale preparation. The liposome association of green fluorescent protein (GFP)-fusion MPs were also tested and recovered as lipid/MP complex after floatation by Accudenz density gradient ultracentrifugation (DGU). Employment of GFP-MPs revealed optimal condition for Accudenz floatation. Using the optimized Accudenz DGU condition, P2RX4/lipid complexes were partially purified and detected as a major band by Coomassie Brilliant Blue (CBB)-staining after SDS-PAGE.

**Conclusion:**

Formation of lipid/AtPPT1 complex during the cell-free synthesis reaction is critical for synthesis of a functional MP. The lipid/MP complex during the translation was observed in all 40 MPs tested. At least 29 MPs, as judged by their higher productivity compared to GFP, might be suitable for a large-scale preparation. MPs synthesized by this method form lipid/MP complexes, which could be readily partially purified by Accudenz DGU. Wheat cell-free protein synthesis in the presence of liposomes will be a useful method for preparation of variety type of MPs.

## Background

MPs comprise up to 30% of genes in fully sequenced genomes and have critical roles in a variety of biological processes including signal transduction, substrate transport, and energy production [1,2]. However, functional and structural studies of MPs are far behind that of soluble proteins. One of the major bottlenecks in the study of MPs is the difficulty in obtaining sufficient amounts of homogeneous protein. For instance, it is typically not easy to purify MPs in preparative scale, due to their low abundance in natural sources. Overexpression of recombinant MPs in living cells is often unsuccessful due to the inhibitory effect of high MP concentration on host cell physiology [3].

Recently, cell-free protein synthesis systems have emerged as a promising tool for MP production [4-6]. In addition to decoupling protein production from the toxic or inhibitory effects on host cell physiology, cell-free systems offer a unique advantage in that protein synthesis can be easily modified by addition of accessory elements, such as detergents and lipids. The addition of detergents and lipids to cell-free systems allows the synthesis of MP/detergent and MP/lipid complexes, respectively, and successful synthesis of functional MPs in this fashion have been reported recently [7-11]. For example, Klammt et al. [12] demonstrated that a G protein-coupled receptor, ETB, can be synthesized in a soluble form using an *Escherichia coli*-based cell-free system supplemented with Brij78, and that the synthesized proteins have ligand binding activity. The ligand binding activity of a human olfactory receptor, hOR17-4, synthesized using a wheat cell-free system in the presence of FC14, has been also reported [13]. Kalmbach et al. [14] reported that *E. coli *cell-free synthesized bacteriorhodopsin in the presence of liposomes was active in black lipid membrane mediated photocurrent measurements. Goren and Fox [15] showed reconstitution of the functional stearoyl Co-A desaturase complex, which consists of three proteins, cytochrome b_5_, cytochrome b_5 _reductase, and human stearoyl-CoA desaturase 1 (hSCD1) synthesized by wheat cell-free system in the presence of asolectin liposomes. However, the general versatility of this method is unclear as the above examples focus on specific MPs.

In a previous study, we reported functional synthesis of a phosphate trranslocator in a wheat cell-free synthesis system supplemented with liposomes and formation of lipid/MP complexes [16]. The mechanism for production of functional protein in this method is not clear, but association of synthesized MP with liposomes may be an important step. To better understand this, we tested the timing of liposome addition to the cell-free MP synthesis reaction. We also investigated whether other MPs synthesized by the method also associates with liposomes. Moreover, we tried to purify the synthesized MP as a lipid/MP complex by DGU.

## Results and Discussion

### Timing of liposome-supplementation to wheat cell-free translation system for synthesis of functional MPs

**Figure 1:**
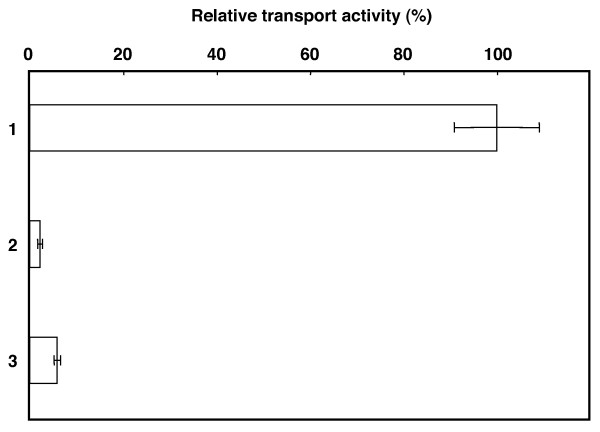
**Effect of timing of liposome-supplementation on transport activity of synthesized AtPPT1**. Three types of AtPPT1 proteins were prepared, 1: AtPPT1 synthesized with liposomes, 2: AtPPT1 synthesized without liposomes, 3: AtPPT1 synthesized without liposomes and mixed with liposomes post-synthesis. Each type of synthesis was reconstituted in liposomes that had been pre-loaded with 30 mM phosphate. Uptake of [^32^P] phosphate into the liposomes was measured. The 100% exchange activity of the synthesized protein was 151 n mol/min/mg proteins. Data is reported as the mean±SD of values from three independent experiments.

Previously, we reported synthesis and liposome association of functional MPs using a wheat cell-free system supplemented with liposomes [16]. To verify that co-translational association of MP with liposomes is critical for functional synthesis, we tested the synthesis of an *Arabidopsis thaliana *phosphate translocator, AtPPT1, in the presence of, absence of, and after post-translational addition of liposomes. These synthesized proteins were reconstituted into liposomes by freeze-thaw and sonication methods after mixing with substrate-preloaded liposomes and phosphate-incorporation activity was measured. Similar to a previous report [16], AtPPT1 synthesized in the absence of liposomes had only 4% of the activity of AtPPT1 synthesized in the presence of liposomes (Figure 1). AtPPT1 synthesized in the absence of liposomes was mixed post-translationally with liposomes and yielded 6% the activity of AtPPT1 synthesized in the presence of liposomes. The association of synthesized AtPPT1 with liposomes, either co- or post-translationally, was measured after sucrose DGU and showed no significant differences (50% co-translational, 62% post-translational). These results indicate that formation of lipid/AtPPT1 complex during the synthesis reaction is an important step for synthesizing functional AtPPT1. Supplementation of liposomes into the cell-free system would prevent aggregation and precipitation of AtPPT1 proteins during synthesis reaction. So, the constitution of AtPPT1/lipid complexes might be effective for formation of functional state MPs in the following freeze-thaw and sonication steps. For preparing MPs in functional state by cell-free system, preventing aggregation and precipitation of MPs during synthesis reaction by supplementation of lipids and/or detergents would be a critical point.

### Cell-free synthesis of MPs in the presence of liposomes

**Figure 2:**
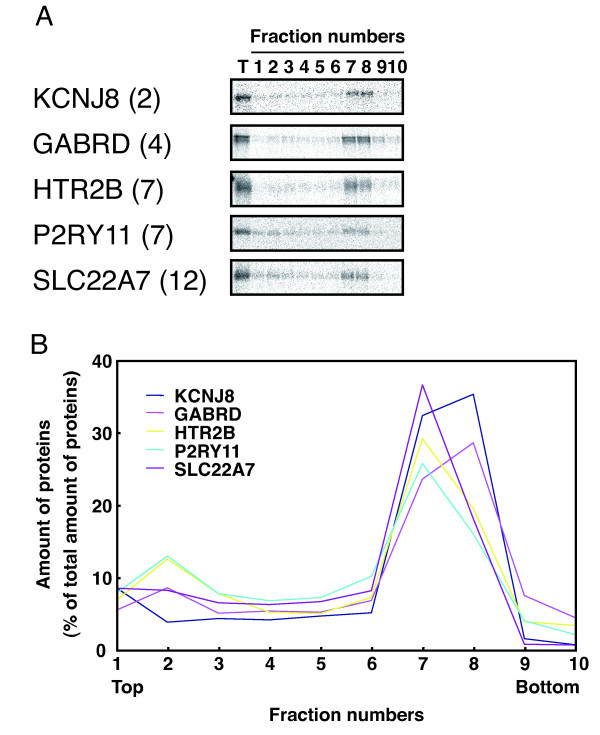
**Association of synthesized MPs with liposomes**. **(A) **SDS-PAGE analysis of liposome association of MPs. MPs were synthesized using a wheat cell-free system supplemented with liposomes and [^14^C]-leucine. After the synthesis reaction, the reaction mixture was subjected to sucrose DGU and fractions were collected from the top of the tube. Synthesized proteins in each fraction were detected by SDS-PAGE and autoradiography. The number of TMDs of each protein is shown in parentheses. T means total fraction of reaction mixture. **(B) **Yield of synthesized proteins in each fraction. After fractionation, synthesized proteins were precipitated by 10% trichloroacetic acid and radioactivity of each fraction was measured by liquid scintillation counter.

Using a wheat cell-free system in the presence of liposomes, a plant MP, AtPPT1, was synthesized as a lipid/MP complex (Figure 1). As MPs account for more than 50% of all human drug targets [17], we wanted to understand if the AtPPT1 membrane association described above was applicable to mammalian MPs. In general, MPs are classified by the number of TMDs and to start we tested five human MPs ranging from 2 to 12 TMD (KCNJ8, 2TMD; GABRD, 4TMD; HTR2B, 7TMD; P2RY11, 7TMD; SLC22A7, 12TMD). The selected mammalian MPs were synthesized in the wheat cell-free system in the presence of asolectin liposomes and their liposome association rates were measured after sucrose DGU. In this experiment, mRNA was prepared from fragments made by split-primer PCR [18,19]. By using a PCR-based fragment as a template for *in vitro *transcription, time consuming steps, such as cloning of a target gene and construction of an expression vector were eliminated. Although the yield of template is low, this step allows for screening large numbers of proteins. As shown in Figure 2, every protein in the test set was associated with liposomes. After sucrose DGU, ^14^C-labeled proteins were predominantly detected in bands six to eight (Figure 2A), which corresponded with the observed liposome bands [16]. For each fraction, the radioactivity was measured and the relative amount of radioactivity for each fraction is depicted in Figure 2B. The extent of association for these proteins ranged from 52 to 73%, when three fractions, numbers six to eight, were treated as liposome fractions. The observed MP association is similar to that seen AtPPT1 (58%). Also there were no significant differences in the extent of association between the five MPs having two to 12 TMDs. These results suggest that association of synthesized MPs with liposomes during wheat cell-free synthesis is as likely to occur in other MPs as was observed for AtPPT1.

**Figure 3:**
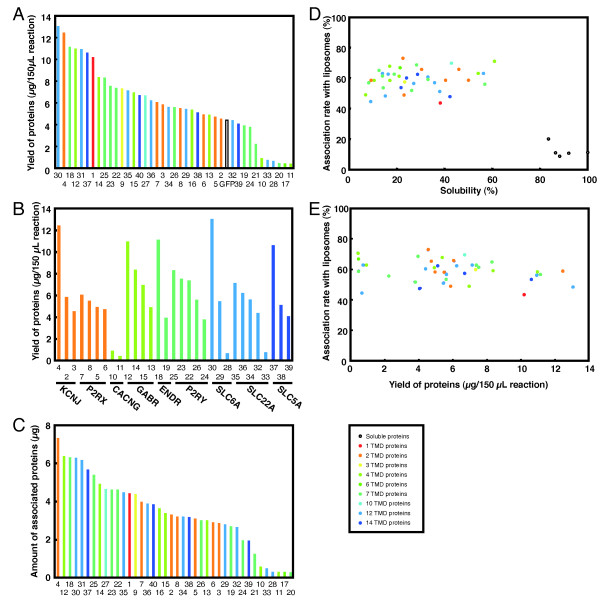
**Yield, extent of liposome association and solubility of each protein**. **(A) **Yield of MPs. MPs were synthesized using the wheat cell-free system supplemented with [^14^C]-leucine and liposomes. Proteins were precipitated by 10% trichloroacetic acid and radioactivity was measured by liquid scintillation counter and the yield of each protein was calculated. The numbers on the x-axis correspond to the proteins in Table I. **(B) **Yield of MPs in each protein family. **(C) **Amount of liposome-associated proteins. MPs were synthesized using the wheat cell-free system supplemented with [^14^C]-leucine and liposomes. After synthesis, the translation reaction was separated by sucrose DGU and fractions were collected from the top of the tube. The protein content of each fraction was estimated through radioactivity measurements of proteins precipitated by 10% trichloroacetic acid. The extent of association was calculated by combining the liposome fractions six through eight. For each protein, the specific amount of association with liposomes was calculated from the synthesis yield and extent of association. **(D) **The relationship between the extent of association and solubility of each protein. Membrane and soluble proteins were synthesized using the wheat cell-free system supplemented with or without liposomes and [^14^C]-leucine. Proteins synthesized without liposomes were separated into soluble and insoluble fraction by centrifugation. The unprocessed synthesis reactions and the insoluble fractions were precipitated by 10% trichloroacetic acid and radioactivity was measured by liquid scintillation counter to determine the solubility for each protein. The extent of association for soluble proteins was calculated using the same method as for MPs. The extent of association and solubility of membrane and soluble proteins are plotted. **(E) **Relationship between extent of association and yield for each protein. Values for the extent of association and yield of each protein are plotted.

Next we further analyzed membrane association using a larger set of proteins, which consisted of 29 human and 6 mouse MPs. While a majority of the test proteins were chosen at random, we ensured that there were multiple representatives of each tested MP family to examine synthesis yield and extent of liposome association within a family. As shown in Figure 3A, the 29 MPs tested showed better synthesis efficiency than GFP (not shown, 4.4 μg/150 μL reaction). The mean value of yield for the set of MPs was 5.9 μg/150 μL reaction. All tested proteins belonging to KCNJ, P2RX, GABR and SC5A families were well synthesized in the wheat cell-free system, whereas production of CACNG family proteins was very low (Figure 3B and Table 1). The remaining proteins, belonging to ENDR, P2RY, SLC6A and SLC22A families had both poorly and well synthesized proteins. (Figure 3B and Table 1).

**Table 1:** Yield, association rate and solubility of synthesized membrane proteins

	Proteins	Anotation	Number of TMD	Molecular weight (kDa)	Yield (* μ*g/150 *μ*l reaction)	Solubility (%)	Association rates with liposomes (%)	Yield × Association rates (* μ*g)
1	Itga1^#^	Integrin	1	131	10.2	38.3	43.3	4.4
2	KCNJ8	Potassium inwardly-rectifying channel	2	48	4.6	22.6	72.6	3.3
3	KCNJ13	Potassium inwardly-rectifying channel	2	41	5.9	23.1	48.7	2.9
4	KCNJ15	Potassium inwardly-rectifying channel	2	43	12.5	10.5	58.8	7.3
5	P2RX1	Purinergic receptor	2	45	4.7	46.0	65.4	3.1
6	P2rx2^#^	Purinergic receptor	2	55	5.0	40.4	58.3	2.9
7	P2RX4	Purinergic receptor	2	43	6.1	30.5	65.4	4.0
8	P2RX5	Purinergic receptor	2	47	5.5	50.0	58.2	3.2
9	GRIA2	Glutamate receptor	3	99	7.3	24.1	59.8	4.4
10	CACNG3	Voltage-dependent calcium channel	4	36	0.9	54.1	62.7	0.6
11	CACNG4	Voltage-dependent calcium channel	4	37	0.4	61.0	70.8	0.3
12	GABRA3	GABA receptor	4	55	11.0	17.1	58.1	6.4
13	GABRB1	GABA receptor	4	54	4.9	21.0	60.9	3.0
14	GABRD	GABA receptor	4	51	8.4	12.5	58.8	4.9
15	GABRG1	GABA receptor	4	54	7.0	6.9	48.6	3.4
16	Glra1^#^	Glycine receptor	4	52	5.4	17.1	67.6	3.6
17	AQP3	Aquaporin	6	32	0.4	21.4	66.6	0.3
18	EDNRA	Endothelin receptor	7	49	11.1	8.3	56.6	6.3
19	EDNRB	Endothelin receptor	7	50	3.9	28.3	68.3	2.7
20	GPR37	G protein-coupled receptor 37	7	67	0.5	33.0	58.8	0.3
21	HTR2B	5-Hydroxytryptamine receptor	7	54	2.2	57.0	55.8	1.2
22	P2RY2	Purinergic receptor	7	42	7.4	18.9	62.4	4.6
23	P2RY10	Purinergic receptor	7	39	7.5	14.0	61.2	4.6
24	P2RY11	Purinergic receptor	7	40	3.8	26.3	51.7	2.0
25	P2RY13	Purinergic receptor	7	38	8.3	12.7	64.8	5.4
26	P2RY14	Purinergic receptor	7	39	5.6	14.3	53.5	3.0
27	SLC1A7	Glutamate transporter	10	61	6.7	42.9	69.4	4.6
28	SLC6A3	Dopamine transporter	12	69	0.7	9.1	44.4	0.3
29	SLC6A13	GABA transporter, GABA	12	68	5.5	38.1	51.1	2.8
30	SLC6A18	Solute carrier family 6, member 18	12	71	13.1	15.3	48.1	6.3
31	Slc18a2^#^	Vesicular monoamine transporter	12	56	10.9	27.1	56.3	6.2
32	SLC22A4	Organic cation transporter	12	62	4.4	33.1	60.2	2.6
33	SLC22A7	Organic anion transporter	12	60	0.8	56.2	62.7	0.5
34	SLC22A8	Organic anion transporter	12	60	5.6	36.0	56.9	3.2
35	SLC22A11	organic anion/cation transporter	12	60	7.1	14.1	62.7	4.5
36	SLC22A12	Organic anion/cation transporter	12	60	6.2	16.4	62.3	3.9
37	Slc5a1^#^	Sodium/glucose cotransporter	14	73	10.6	21.8	53.4	5.7
38	SLC5A6	Sodium-dependent vitamin transporter	14	69	5.1	28.7	62.1	3.2
39	SLC5A10	Sodium/glucose cotransporter	14	62	4.1	42.2	47.5	1.9
40	Slc7a1^#^	Cationic amino acid transporter	14	67	6.7	23.3	57.3	3.8

^# ^Mouse clone

The extent of association of all 40 proteins tested, containing between one and 14 TMDs, were from 43 to 73% and the mean value was 59% (Table 1). The amount of proteins associated with liposomes was calculated from the yield and extent of association for each protein (Figure 3C and Table 1). The mean value of the association yield was 3.4 μg/150 μL reaction. When analyzing association by the number of TMDs, the lowest extent of association was 43% for 1 TMD (Itga1), whereas the mean value of association for proteins having more than 2 TMDs was approximately 60% (Table 1). These results indicate that efficiency of association of proteins having only 1TMD might be lower than that of proteins having more than 2TMD.

Figure 3D shows the relationship between solubility and association rate of tested proteins. Solubility of MPs synthesized using the wheat cell-free system in the absence of liposomes ranged from eight to 61%. The extent of association of these proteins ranged from 40 to 70% regardless of their solubilities. On the other hand, soluble proteins showed more than 80% solubility and their association rates were less than 20%, indicating that the extent of liposome association for soluble proteins is much lower than that of MPs. In comparison to the tested soluble proteins, the MPs examined had a wide variety of sizes, functional roles and topologies, and all of them appeared to be efficiently associated with liposomes during cell-free synthesis. Moreover, there were no significant relationship between the extent of protein association and their respective yields (Figure 3E).

In this experiment, we found that a variety of MPs make complexes with lipids during wheat cell-free synthesis in the presence of liposomes. It is not yet clear whether these MPs are integrated into liposomes or attached to surface of liposomes with their hydrophobic regions. For replying to this question, further experiments for evaluating function and/or structure of each synthesized MP are needed. However, as association of MPs with liposomes prevents aggregation and precipitation of synthesized MPs during synthesis reaction, formation of lipid/MP complex would be an important step for preparing MPs.

Katzen et al. [20] recently reported that insertion of EmrE into a discoidal membrane scaffold in correctly folded state during cell-free synthesis by analysis of binding activity of its substrate, tetraphenylphosphonium. Moritani et al. [21] demonstrated that connexin-43 synthesized by an *E. coli *cell-free system, PURE system containing minimum protein synthesis factors [22], in the presence of liposomes is directly integrated with a uniform orientation into liposome membrane. The connexin-43 synthesized into liposomes was shown to deliver a hydrophilic and bioactive oligo-peptide to cells through gap junctions [23]. They speculate that liposomes may have chaperone-like function because their system consists of only protein synthesis factors and liposomes [21]. Although it is still not clear any kinds of MPs can receive such a chaperone-like effect from liposomes, the addition of artificial membranes to cell-free reactions appears to be effective in synthesizing membrane-stabilized MPs.

### Partial purification of synthesized MPs by density-gradient ultracentrifugation

In this study, we demonstrated that a variety of mammalian MPs are efficiently associated with liposomes during wheat cell-free synthesis supplemented with liposomes. Next, we tried to partially purify the synthesized lipid/MP complexes from the endogenous wheat germ proteins. For this experiment we used P2RX4 as a model protein, because both the yield (6.1 μg) and extent of association (66%) are very close to the mean value for both of these parameters in the full mammalian MP dataset above (5.9 μg and 59%, respectively).

**Figure 4:**
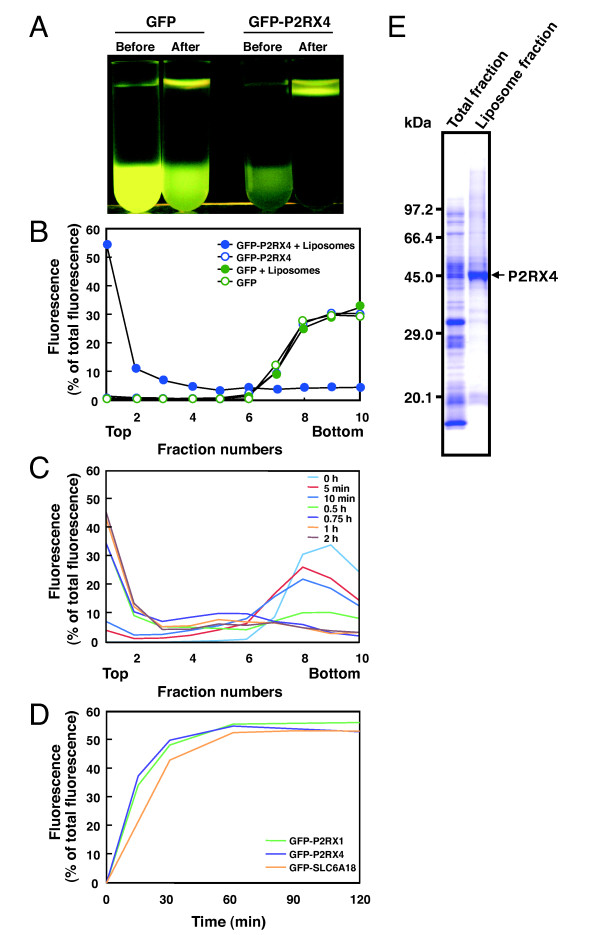
**Partial purification of P2RX4 proteins by Accudenz DGU**. **(A) **Floatation of GFP-P2RX4/lipid complex by Accudenz DGU. GFP and GFP-P2RX4 were synthesized using the wheat cell-free system supplemented with liposomes. After the synthesis reaction, the reaction mixture was subjected to Accudenz DGU. Fluorescence images were visualized using a transilluminator. **(B) **Yield of synthesized protein in each fraction. After DGU, fractions were collected from the top of the tube and the fluorescence intensity of each fraction was measured with a spectrofluorometer. **(C) **Effect of centrifugation time on floatation of lipid/MP complex. Synthesized GFP-fusion MPs were subjected to Accudenz DGU at varying centrifugation times. After DGU, fractions were collected from the top of the tube and the fluorescence intensity of each fraction was measured using a spectrofluorometer. **(D) **The effect of centrifugation time on the floatation of liposomes containing different MPs. GFP-P2RX4, -P2RX1, and -SLC6A18 fusion proteins were used in this experiment. Fraction No. one was treated as the liposome fraction and its fluorescence intensity in each condition was plotted. **(E) **Partial purification of P2RX4 proteins. P2RX4 was synthesized using the wheat cell-free system supplemented with liposomes. After the synthesis reaction, the reaction mixture was concentrated and subjected to Accudenz DGU. The synthesis mixture and the top lipid/MP complex fraction after DGU were applied to SDS-PAGE and stained by CBB.

A GFP-P2RX4 fusion protein was synthesized using the wheat cell-free system supplemented with liposomes and the resultant lipid/MP complexes were subjected to Accudenz DGU. Accudenz is a non-toxic medium used for fractionation of proteins, organelles, and cells [15,24-26]. The wheat cell-free synthesis reaction was brought to 40% Accudenz by addition of an 80% Accudenz solution, placed at the bottom of an ultra-centrifugation tube, and overlaid with 35% Accudenz solution, 30% Accudenz solution and DGU solution. It is expected that after Accudenz DGU, liposomes float to the top of the centrifuge tube owing to their low density [15,24]. As shown in Figure 4A, the fluorescence from GFP-P2RX4 fusion proteins was observed at the top of the gradient after Accudenz DGU, while the majority of the fluorescence from a soluble GFP control remained at the bottom. After fractionation, the fluorescence of each fraction was measured with a spectrofluorometer (excitation 480 nm/emission 510 nm). High fluorescence intensity at the top fraction, associated with floated liposomes, was detected in GFP-P2RX4 sample synthesized by liposome supplemented wheat cell-free synthesis (Figure 4B). In the other samples, GFP synthesized with or without liposomes and GFP-P2RX4 synthesized without liposomes, fluorescence was mainly detected in the lower fractions (Figure 4B). The extent of association of GFP-P2RX4 in this experiment (66%: Fraction 1 and 2) was similar to the association of P2RX4 calculated by sucrose DGU (66%). Although we also tested association of P2RX2-GFP with liposomes with same procedure, there were no significant differences in association rate (data not shown).

Although fluorescence was seen at the top of the gradient in the soluble GFP control after Accudenz DGU by using a transilluminator (excitation: 400-500 nm) with a filter for removing blue light (Figure 4A), the fluorescence in the top fraction was not detected with a spectrofluorometer using a condition for detection of fluorescence from GFP (excitation 480 nm/emission 510 nm) (Figure 4B). These results indicate that the fluorescence in the top fraction is not attributed to GFP but probably to the liposomes. In comparison to sucrose DGU, Accudenz DGU makes lipid/MP complex recovery easier due to the fact that liposomes float in Accudenz as opposed to sinking as in sucrose. Additionally, Accudenz is a preferred DGU solution because it is non-toxic.

The GFP-fusion P2RX4 is an ideal construct to determine the optimal conditions for Accudenz DGU as the liposome-associated protein can be readily monitored by fluorescence. We tested several centrifugation times for Accudenz DGU. As shown in Figure 4C, the fluorescence in the top fraction gradually increased in proportion centrifugation time. One-h centrifugation appears to be sufficient for lipid/MP complex floatation. The results from Accudenz DGU of two additional MPs, GFP-P2RX1 and GFP-SLC6A18, also showed that the floatation rates of lipid/MP complex plateaus at a one-h centrifugation (Figure 4D).

As shown in Figure 4B, GFP and GFP-P2RX4 could be clearly separated by DGU. The result suggests that lipid/MP complexes can be separated from endogenous proteins derived from the wheat germ extract by Accudenz DGU. Nomura et al. also reported that cytochrome b5 synthesized by the wheat cell-free system in the presence of liposomes was easily purified by simplified discontinuous DGU [27]. In addition, Goren and Fox showed that a MP, hSCD1, synthesized by the wheat cell-free system supplemented with liposomes could be separated from endogenous wheat germ proteins by Accudenz DGU [15]. We next tried to purify P2RX4, without a GFP fusion, by this method. After synthesis supplemented with liposomes, lipid/MP complexes were concentrated by centrifugation and applied to Accudenz DGU. After ultracentrifugation, the top fraction was recovered and applied to SDS-PAGE. As shown in Figure 4E, P2RX4 was detected as a major band by CBB-staining after SDS-PAGE. This result indicates that lipid/MP complexes synthesized by our wheat cell-free system supplemented with liposomes can be partially purified by Accudenz DGU. To test whether proteins purified by this method are functional, we tested the activity of AtPPT1 partially purified as lipid/MP complexes. We detected transport activity of AtPPT1 purified with Accudenz DGU (data not shown). In addition to functional analysis, this partially purified protein could be used for structural analysis. However, some contaminating proteins from wheat germ extracts do float with the lipid/MP complexes. Goren and Fox [15] reported that, along with other unclassified contaminants, HSP70, elongation factor 1 α and 16.9 kDa heat shock protein were seen in wheat cell-free synthesized and floated lipid/MP complexes. For functional and structural analysis, further purification steps would be required including affinity-tag purification and gel-filtration.

## Conclusion

We have developed a production method for lipid/MP complexes using a wheat germ cell-free system supplemented with liposomes. Using this method, a variety of mammalian MPs were efficiently associated with liposomes co-translationally. The resultant lipid/MP complexes are easily separated from other proteins in wheat germ extract by DGU. This synthesis method is useful in the preparation of MP for structural and functional analysis.

## Methods

### Wheat Cell-free protein synthesis

Details of the wheat cell-free reaction were described in previously [28-30]. The 40 MPs and five soluble proteins in this study were selected from the Mammalian Gene Collection and FANTOM collection (Danaform, Yokohama, Japan). The unique primers for each protein (Additional file 1 Table S1) were designed and templates for transcription were made by the split-primer PCR method as described previously [18,19]. The first PCR was performed with 10 nM of the gene specific primer (Additional file 1 Table S1) and 10 nM of the AODA2303 primer (5'-GTCAGACCCCGTAGAAAAGA) or 10 nM AODS primer (5'-TTTCTACGGGGTCTGACGCT). The second PCR was amplified with 100 nM SPu primer (5'-GCGTAGCATTTAGGTGACACT), 1 nM deSP6E01-S1 primer (5'-GGTGACACTATAGAACTCACCTATCTCCCCAACACCTAATAACATTCAAT CACTCTTTCCACTAACCACCTATCTACATCACCAACCACCCACCACCACCAATG), and 100 nM AODA2303 primer or 100 nM AODS primer. mRNA was prepared by *in vitro *transcription in a reaction volume of 100 * μ*l and was purified by ethanol precipitation. The mRNA pellet was resuspended in 30 * μ*l of water. The translation reaction was performed using the bilayer method, supplemented with [^14^C-leucine (50 * μ*Ci/ml, GE Healthcare, Tokyo, Japan) [31], in which a 25 * μ*l translation layer was overlaid with a 125 * μ*l substrate feeding buffer. Asolectin liposomes were prepared as described previously [16,32] and added to both layers (10 mg/ml final concentration). The bilayer reaction was incubated at 26°C for 16 h. After the reaction, the amount of [^14^C]-leucine incorporation into synthesized proteins, an indicator of synthesis yield, was determined by 10% trichloroacetic acid precipitation and liquid scintillation spectroscopy.

### Sucrose density gradient ultracentrifugation

Lipid/MP complexes were separated from proteins in the wheat germ extract by sucrose DGU. Synthesized proteins (100 μl) were loaded onto a discontinuous sucrose gradient consisting of 1,300 μl of 10% (w/v) and 600 μl of 30% (w/v) sucrose in DGU solution containing 140 mM NaCl, 5.4 mM KCl and 10 mM Tris-HCl (pH 8.0). The gradient was centrifuged at 105,000 *g *for 4 h at 4°C in a Hitachi S55S rotor (Hitachi High-Technology, Tokyo, Japan). Fractions were collected from the top of the tubes, and the protein content of each fraction was estimated by measurement of the amount of radioactivity associated with proteins precipitated by 10% trichloroacetic acid.

### Transport assay of phosphate translocator

Liposomes were prepared from acetone-washed asolectin (Sigma-Aldrich, Tokyo, Japan) by sonication for 5 min at 4°C in water. AtPPT1 was synthesized by wheat cell-free system with (10 mg/ml final concentration) or without liposomes. Half of the AtPPT1 synthesized without liposomes was mixed with liposomes (10 mg/ml final concentration) after the synthesis reaction and incubated for 30 min at 26°C. These reaction mixtures were desalted by gel filtration with a MicroSpin G-25 column (GE Healthcare) that had been equilibrated with 10 mM Tricine-KOH (pH 7.6).

The amount of protein synthesized in the cell-free system was estimated from the incorporation of [^14^C]-leucine. After the reaction, the amount of [^14^C]-leucine incorporation into synthesized proteins, an indicator of synthesis yield, was determined by 10% trichloroacetic acid precipitation and liquid scintillation spectroscopy.

Substrate-including liposomes (80 mg/ml final concentration) were prepared from acetone-washed asolectin by sonication for 5 min at 4°C in a solution containing 200 mM Tricine-KOH (pH 7.6), 40 mM potassium gluconate and 60 mM potassium phosphate (substrate). Desalted reaction mixtures were mixed with the substrate-preloaded liposomes, frozen in liquid nitrogen, thawed at room temperature and sonicated for 18 sec. Substrate that remained outside of the liposomes was removed with a Dowex AG-1X8 column (Bio-Rad, Tokyo, Japan) that had been equilibrated with a solution containing 100 mM sodium gluconate, 40 mM potassium gluconate and 10 mM Tricine-KOH (pH 7.6). The liposome mixture was applied to the column and eluted with the equilibration solution.

Transport reactions were initiated by the addition of 13 μl of [^32^P] Pi (GE healthcare) to 300 μl of liposomes (final phosphate concentration, inside: 30 mM, outside: 0.5 mM). The assay was performed at 25°C for 2 min, and the reaction was terminated by application of the reaction mixture to a Dowex AG-1X8 column that had been equilibrated with 150 mM sodium acetate. The radio-activity associated with the eluted liposomes was measured with a liquid scintillation spectrometer.

### Plasmid construction

GFP fragments were amplified by PCR with the primers EcoRV-GFP-5' (5'-GAGAGATATCATGGGCCTGAACGACATCTTCGAGGCCCAGAAGATCGAG TGGCACGAAGGTGGAGGTGGAATGGTGAGCAAGGGCGAGGA-3') and GFP-NotI-3' (5'-TCTCGCGGCCGCTCCACCTCCACCCTTGTACAGCTCGTCCATGC-3). The PCR fragments were digested with *Eco*RV and *Not*I and then cloned into the corresponding sites in the pEU-E01-MCS vector (Cellfree Sciences, Matsuyama, Japan). The resultant plasmid was designated pEU-E01-GFP-N. P2RX4, P2RX1, and SLC6A18 were obtained from cDNA clones and amplified by PCR with the primer sets NotI-P2RX4-5' (5'-GAGAGCGGCCGCTGAAAACCTGTATTTTCAGGGCATGGCGGGCTGCTGC GCCGC) and P2RX4-SalI-3' (5'-AGAGGTCGACTCACTGGTCCAGCTCACTAG), NotI-P2RX1-5' (5'-GAGAGCGGCCGCTGAAAACCTGTATTTTCAGGGCATGGCGGGCTGCTGC GCCGC) and P2RX1-SalI-3' (5'-AGAGGTCGACTCACTGGTCCAGCTCACTAG), and NotI-SLC6A18-5' (5'-GAGAGCGGCCGCTGAAAACCTGTATTTTCAGGGCATGGCTCATGCCCCA GAACC) and SLC6A18-SalI-3' (5'-AGAGGTCGACTCAGCGCATGTCCGTGTCCG), respectively. These PCR fragments were digested with *Not*I and *Sal*I and then cloned into the *Not*I-*Sal*I sites of pEU-E01-GFP-N. The resultant plasmids were designated pUE-E01-GFP-P2RX4, pUE-E01-GFP-P2RX1, and pUE-E01-GFP-SLC6A18, respectively, and used for production of GFP-fusion proteins. The P2RX4 ORF was amplified by PCR using the primer pair P2RX4-EcoRV-5' (5'-GAGAGATATCATGGCGGGCTGCTGCGCCGC) and P2RX4-NotI-3' (5'-CTCTGCGGCCGCTCCACCTCCACCCTGGTCCAGCTCACTAGCAA). The PCR product was digested with *Eco*RV and *Not*I and then inserted into *Eco*RV-*Not*I sites of pEU-E01-MCS. The resultant plasmid was designated pEU-E01-P2RX4. The nucleotide sequences of each DNA fragment amplified by PCR was confirmed by DNA sequencing.

### Detection and quantification of fluorescence from GFP proteins

Fluorescence images were visualized with a transilluminator (excitation: 400-500 nm, Dark Reader DR45M, Clare Chemical Research, Dolores, CO). Fluorescence intensity from GFP proteins was measured by a, Wallac 1420 Multilabel Counter spectrofluorometer (Perkin-Elmer Japan, Chiba, Japan).

### Partial purification of P2RX4

mRNA was prepared by *in vitro *transcription using pEU-E01-P2RX4 as the template. The translation reaction was performed in the presence of liposomes (10 mg/ml final concentration) using the bilayer method with a bottom layer of 500 μl and an upper layer of 5500 μl. After the synthesis reaction, the reaction mixture was concentrated to 300 μl with a concentrator (Amicon Ultra-15, 30,000 MWCO, Millipore-Japan, Tokyo, Japan). For Accudenz (Accurate Chemical and Scientific, Westbury, NY) DGU, Accudenz was dissolved into DGU solution to make 30, 35, and 80% (w/v) Accudenz solutions. Three hundred μl of concentrated sample was mixed with 300 μl of 80% (w/v) Accudenz solution. The resultant 40% Accudenz solution containing the synthesized protein was placed in the bottom of a centrifuge tube, and overlaid with 650 μl of 35% (w/v) Accudenz solution, 650 μl of 30% (w/v) Accudenz solution, and 100 μl of DGU solution. The gradient was centrifuged at 105,000 *g *for 4 h at 4°C in a Hitachi S55S rotor (Hitachi High-Technology, Tokyo, Japan).

## Abbreviations

CBB: Coomassie Brilliant Blue; DGU: density gradient ultracentrifugation; hSCD1: human stearoyl-CoA desaturase 1; Liposomes: exogenous liposomes; MP: membrane protein; PCR: polymerase chain reaction; SDS-PAGE: sodium dodecyl sulfate-polyacrylamide gel electrophoresis; TMD: transmembrane domain

## Authors' contributions

YE and TS designed the experiment. AN, TO, SM, and TI performed the experiments.AN and TS wrote the manuscript. All authors read and approved the final manuscript.

## Supplementary Material

Additional file 1 Table S1. Click here for file
