# Supplementary material for: Production and partial purification of membrane proteins using a liposome-supplemented wheat cell-free translation system
Source: BMC Biotechnol. 2011 Apr 11;11:35. doi: 10.1186/1472-6750-11-35 (PMC3090341; doi:10.1186/1472-6750-11-35)
Supplement: Additional file 1 — Table S1. [file 1472-6750-11-35-S1.DOC]

# Table S1: Sequence of primers for template amplification

| Proteins | Sequence |
| --- | --- |
| Membrane proteins | |
| Itga1 | CCACCCACCACCACCAATGGTCCCCAGGCGTCCTG |
| KCNJ8 | CCACCCACCACCACCAATGTTGGCCAGAAAGAGTA |
| KCNJ13 | CCACCCACCACCACCAATGGACAGCAGTAATTGCA |
| KCNJ15 | CCACCCACCACCACCAATGGATGCCATTCACATCG |
| P2RX1 | CCACCCACCACCACCAATGGCACGGCGGTTCCAGG |
| P2rx2 | CCACCCACCACCACCAATGGCCGCTGCACAGCCCC |
| P2RX4 | CCACCCACCACCACCAATGGCGGGCTGCTGCGCCG |
| P2RX5 | CCACCCACCACCACCAATGGGGCAGGCGGGCTGCA |
| GRIA2 | CCACCCACCACCACCAATGCAAAAGATTATGCATA |
| CACNG3 | CCACCCACCACCACCAATGAGGATGTGTGACAGAG |
| CACNG4 | CCACCCACCACCACCAATGGTGCGATGCGACCGCG |
| GABRA3 | CCACCCACCACCACCAATGATAATCACACAAACAA |
| GABRB1 | CCACCCACCACCACCAATGTGGACAGTACAAAATC |
| GABRD | CCACCCACCACCACCAATGGACGCGCCCGCCCGGC |
| GABRG1 | CCACCCACCACCACCAATGGGTCCTTTGAAAGCTT |
| Glra1 | CCACCCACCACCACCAATGTACAGCTTCAATACTC |
| AQP3 | CCACCCACCACCACCAATGGGTCGACAGAAGGAGC |
| EDNRA | CCACCCACCACCACCAATGGAAACCCTTTGCCTCA |
| EDNRB | CCACCCACCACCACCAATGCAGCCGCCTCCAAGTC |
| GPR37 | CCACCCACCACCACCAATGCGAGCCCCGGGCGCGC |
| HTR2B | CCACCCACCACCACCAATGGCTCTCTCTTACAGAG |
| P2RY2 | CCACCCACCACCACCAATGGCAGCAGACCTGGGCC |
| P2RY10 | CCACCCACCACCACCAATGGCTAACCTTGACAAAT |
| P2RY11 | CCACCCACCACCACCAATGGCAGCCAACGTCTCGG |
| P2RY13 | CCACCCACCACCACCAATGAACACCACAGTGATGC |
| P2RY14 | CCACCCACCACCACCAATGATCAATTCAACCTCCA |
| SLC1A7 | CCACCCACCACCACCAATGGTGCCGCATGCCATCT |
| SLC6A3 | CCACCCACCACCACCAATGAGTAAAAGCAAATGCT |
| SLC6A13 | CCACCCACCACCACCAATGGATAGCAGGGTCTCAG |
| SLC6A18 | CCACCCACCACCACCAATGGCTCATGCCCCAGAAC |
| Slc18a2 | CCACCCACCACCACCAATGGCCCTGAGCGATCTGG |
| SLC22A4 | CCACCCACCACCACCAATGCGGGACTACGACGAGG |
| SLC22A7 | CCACCCACCACCACCAATGGGCTTTGAGGAGCTGC |
| SLC22A8 | CCACCCACCACCACCAATGACCTTCTCGGAGATCC |
| SLC22A11 | CCACCCACCACCACCAATGGCGTTCTCGAAGCTCT |
| SLC22A12 | CCACCCACCACCACCAATGGCATTTTCTGAACTCC |
| Slc5a1 | CCACCCACCACCACCAATGGGACAGTAGCACCTTGA |
| SLC5A6 | CCACCCACCACCACCAATGAGTGTAGGGGTGAGCA |
| SLC5A10 | CCACCCACCACCACCAATGACGTGGTGGCCGATTG |
| Slc7a1 | CCACCCACCACCACCAATGGGCTGCAAAAACCTGC |
|  |  |
| Soluble proteins | |
| Gart | CCACCCACCACCACCAATGGCAGCCCGAGTTCTTG |
| Dscr1 | CCACCCACCACCACCAATGGAGGACGGCGTGGCCG |
| Pyp | CCACCCACCACCACCAATGAGCGGCTTCAGCAGCG |
| Sod1 | CCACCCACCACCACCAATGGCGATGAAAGCGGTGT |
| Cbr3 | CCACCCACCACCACCAATGTCGTCCTGCAGCCGCG |
